# Human protein aging: modification and crosslinking through dehydroalanine and dehydrobutyrine intermediates

**DOI:** 10.1111/acel.12164

**Published:** 2013-11-19

**Authors:** Zhen Wang, Brian Lyons, Roger J W Truscott, Kevin L Schey

**Affiliations:** 1 Department of Biochemistry and Mass Spectrometry Research Center, Vanderbilt University School of MedicineNashville, TN, 37232, USA; 2 Save Sight Institute, University of SydneySydney, NSW, 2000, Australia; 3 Illawarra Health and Medical Research Institute, University of WollongongWollongong, NSW, 2522, Australia

**Keywords:** aging, glutathione, lens, protein–protein crosslinking

## Abstract

Nonenzymatic post-translational modification (PTM) of proteins is a fundamental molecular process of aging. The combination of various modifications and their accumulation with age not only affects function, but leads to crosslinking and protein aggregation. In this study, aged human lens proteins were examined using HPLC–tandem mass spectrometry and a blind PTM search strategy. Multiple thioether modifications of Ser and Thr residues by glutathione (GSH) and its metabolites were unambiguously identified. Thirty-four of 36 sites identified on 15 proteins were found on known phosphorylation sites, supporting a mechanism involving dehydroalanine (DHA) and dehydrobutyrine (DHB) formation through β-elimination of phosphoric acid from phosphoserine and phosphothreonine with subsequent nucleophilic attack by GSH. *In vitro* incubations of phosphopeptides demonstrated that this process can occur spontaneously under physiological conditions. Evidence that this mechanism can also lead to protein–protein crosslinks within cells is provided where five crosslinked peptides were detected in a human cataractous lens. Nondisulfide crosslinks were identified for the first time in lens tissue between βB2- & βB2-, βA4- & βA3-, γS- & βB1-, and βA4- & βA4-crystallins and provide detailed structural information on *in vivo* crystallin complexes. These data suggest that phosphoserine and phosphothreonine residues represent susceptible sites for spontaneous breakdown in long-lived proteins and that DHA- and DHB-mediated protein crosslinking may be the source of the long-sought after nondisulfide protein aggregates believed to scatter light in cataractous lenses. Furthermore, this mechanism may be a common aging process that occurs in long-lived proteins of other tissues leading to protein aggregation diseases.

## Introduction

Long-lived proteins are subject to numerous post-translational modifications including racemization, oxidation, glycation, truncation, and deamination. (Robinson & Robinson, 2001; Stadtman, 2001; Soskić *et al*., 2008; Jaisson & Gillery, 2010; Truscott, 2011). These modifications often result in functional deficits and have been causally implicated in diseases such as diabetes mellitus, Alzheimer’s disease, atherosclerosis (Jaisson & Gillery, 2010) and may ultimately lead to reduced lifespan (Truscott, 2011). Among the longest-lived proteins in the body are proteins of the ocular lens. Remarkably, there is no significant protein turnover in differentiated lens fiber cells; therefore, the center of the lens contains proteins as old as the organism (Lynnerup *et al*., 2008), and this fact makes it a convenient tissue to study age-related post-translational modifications.

With age, a number of biochemical and biophysical changes occur in the lens and extensive studies have been conducted to elucidate age-related changes in human lens proteins. Besides lens protein truncation (Lampi *et al*., 1998; Grey & Schey, 2009), significant changes to lens proteins include protein aggregation leading to an increase in high molecular weight complexes and a reduction in protein solubility (Harrington *et al*., 2004). In age-related nuclear cataract lenses, a significant amount of high molecular weight complex remains even after the tissue has been treated with denaturing and reducing agents, suggesting the formation of nondisulfide covalent protein–protein crosslinks (Dilley & Pirie, 1974; Srivastava *et al*., 2004). It is thought that protein aggregation, crosslinking, and insolubilization processes contribute to the development of age-related lens opacity (Dilley & Pirie, 1974). Similar protein aggregation processes are also common features of aging of other long-lived postmitotic cells such as neurons, retinal pigment epithelium, cardiac myocytes, and skeletal muscle fibers (Grune *et al*., 2004; Goebel & Blaschek, 2011; Polymenidou & Cleveland, 2011). Understanding the factors that lead to protein–protein crosslinking is critically important in elucidating the aging process and in identifying disease-specific crosslinks. Evidence suggests that a variety of post-translational modifications and oxidative stress could contribute to protein–protein aggregation and crosslinking. Several crosslinking mechanisms have been proposed based on the identification of crosslinked compounds such as advanced glycation end products (Nagaraj *et al*., 1991), γ-glutamyl-ε-lysine (Lorand *et al*., 1981), lanthionine (LAN), histidinoalanine (HAL), and lysinoalanine (LAL) (Kanayama *et al*., 1987; Linetsky *et al*., 2004; Linetsky & LeGrand, 2005); however, the exact proteins and sites of crosslinking remain unknown.

Lanthionine, HAL, and LAL are crosslinks formed through a dehydroalanine (DHA) intermediate. DHA is detected in food proteins that have been treated with heat or alkali (Sen *et al*., 1977). The formation of DHA results from a hydroxide ion-induced β-elimination reaction, a process that can also occur in long-lived proteins under physiological conditions as evidenced by the detection of DHA in tissues such as human lens (Srivastava *et al*., 2004) and dentin (Masters, 1985; Cloos & Jensen, 2000). The amino acid residues that can undergo decomposition to DHA include cysteine (Bar-Or *et al*., 2008), serine, and phosphoserine (Sen *et al*., 1977; Cloos & Jensen, 2000). DHA is reactive and is subject to nucleophilic attack by cysteine, histidine, and lysine to form LAN, HAL, and LAL, respectively. Although free amino acids in tissues can add to DHA, if lysine, histidine, or cysteine residues react with DHA, this reaction can lead to intramolecular and intermolecular protein–protein crosslinks (Bessems *et al*., 1987; Cloos & Jensen, 2000; Linetsky *et al*., 2004). LAN, HAL, and LAL have been detected in dentin (Cloos & Jensen, 2000) as well as in normal and cataractous lenses, with the level of these potential crosslinks being significantly higher in cataractous lenses (Bessems *et al*., 1987; Linetsky *et al*., 2004). Previous identification of LAN, HAL, and LAL in aged tissues was based on the analysis of acid-hydrolyzed samples; however, heat increases the formation of DHA (Samuel & Silver, 1963) and the subsequent nucleophilic addition can also be catalyzed by acid (Singh & Goswami, 2008), which raises a concern of artifactual crosslink formation induced by acid hydrolysis. In addition, acid hydrolysis removes any information of which proteins, if any, are involved in the crosslinking process. Detailed information of which proteins are crosslinked and how they are crosslinked is essential to distinguish normal aging and disease processes. In addition to being a key participant in protein–protein crosslinking, DHA is expected to react with free thiols and amines to produce covalently modified proteins. Linetsky *et al*. reported glutathionylation of lens proteins via a DHA intermediate using ELISA (Linetsky & LeGrand, 2005); however, this method could not identify the proteins or amino acids involved.

In the present study, we identified polypeptides that are modified by glutathione, cysteine, and glycine–cysteine dipeptide as well as homocysteine through thioether linkages. Our findings support a mechanism involving glutathione addition to a DHA residue. If DHA were indeed being formed, it could also act to crosslink proteins and additional results unambiguously confirmed DHA-mediated protein–protein crosslinks in aged lens proteins. Our results, for the first time, identify the sites in human proteins that are modified by free thiol compounds through thioether bonds and also elucidate the sites of novel covalent crosslinking of proteins in a cataractous lens. Our results provide direct evidence for a mechanism involving spontaneous formation of DHA and DHB intermediates from phosphoserine, phosphothreonine as well as cysteine, leading to protein modification and protein–protein crosslinking. The methods used in this study can be used as a guide to identify similar modifications and protein–protein crosslinks in long-lived proteins from other tissues.

## Results

**Figure 1:**
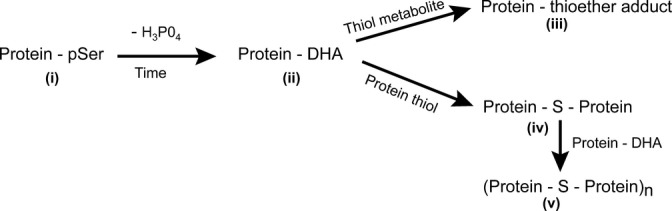
Diagram of reactions and products formed in human lens tissue with age.

Figure 1 indicates the predicted reactions leading to observed products characterized below.

### Identification of modifications on Ser and Thr in the normal human lens

**Figure 2:**
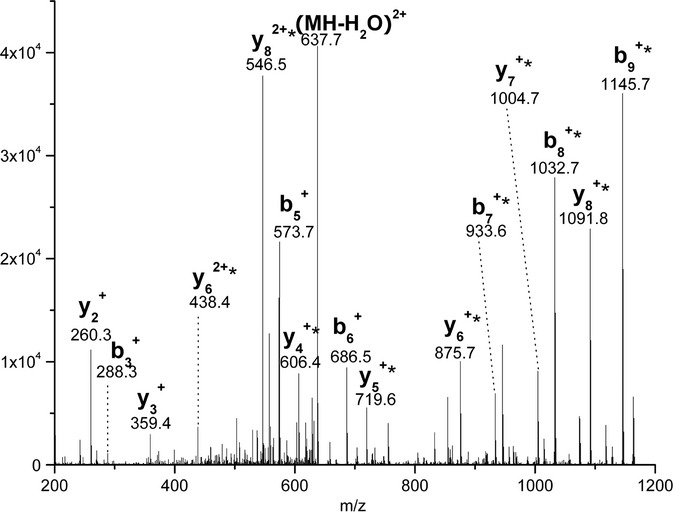
Tandem mass spectrum of AQP0 peptide 229-238 (SISERLSVLK) with S235 modified by Cys-Gly dipeptide. [MH]^2+^_mono_ = 646.3549. b- and y-ions are labeled, and asterisks indicate fragment ions with modification.

**Figure 3:**
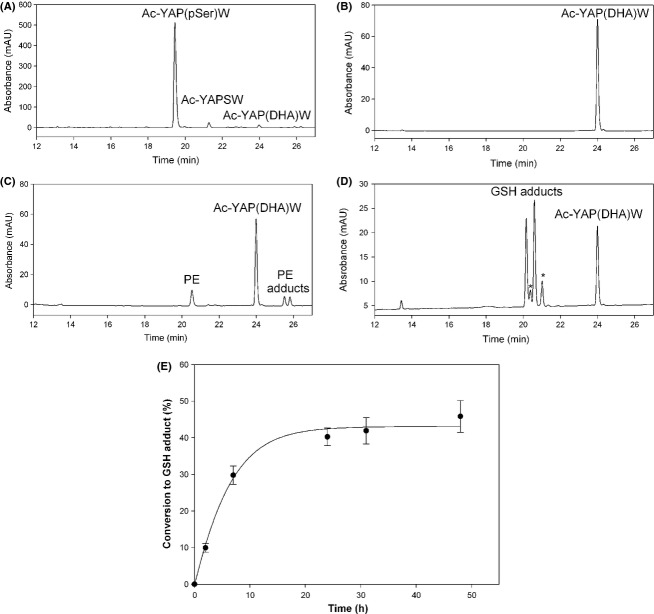
Formation of DHA from a phosphoserine (pSer) peptide and the subsequent addition of GSH and PE. HPLC traces showing: (A) the formation of Ac-YAPSW and Ac-YAP(DHA)W following incubation of Ac-YAP(pSer)W in 100 mm phosphate buffer pH 7.4 for 15 days at 60 °C, (B) Ac-YAP(DHA)W isolated from the above incubation and characterized by NMR (Fig. S3), (C) Products formed when Ac-YAP(DHA)W was incubated in 100 mm phosphate buffer pH 7.4 for 24 h at 37 °C with a tenfold molar excess of phenylethylamine (PE), (D) Products formed when Ac-YAP(DHA)W was incubated under the same conditions with GSH. Cys-Gly adducts are indicated by an asterisk. (E) Time course of GSH adduct formation from Ac-YAP(DHA)W. Ac-YAP(DHA)W was incubated at 37 °C with a tenfold molar excess of GSH in 100 mm phosphate buffer pH 7.4, and adduct formation was monitored over time by HPLC. Detection was at 280 nm.

Multidimensional LC-MS/MS analyses of trypsin-digested lens membrane fractions analyzed by TagRecon using a blind modification search revealed a 160 amu addition to Ser residues on lens proteins such as AQP0, MP20, and several crystallins. A representative tandem mass spectrum is shown in Fig. 2 for modified AQP0 229-238. The tandem mass spectrum clearly indicates that the modification is on a single Ser residue (Fig. 2), ruling out the possibility of phosphorylation at two distinct sites. Moreover, the accurate mass of the modified peptide obtained from the Orbitrap mass spectrometer does not match the predicted peptide masses for diphosphorylation or pyrophosphorylation within the instrument mass accuracy of < 5 ppm. The mass addition of 160.0288 amu suggests modification by a Cys-Gly dipeptide through a thioether bond (peptide mass error = 1.04 ppm). The most likely origin of Cys-Gly is from the breakdown of GSH based on *in vitro* results shown in Fig. 3 (discussed below). Based on the identification of a thioether modification, all tandem mass spectra were re-searched for differential modifications on Ser and Thr residues by thiol-containing molecules such as GSH, Cys, Cys-Gly, and homocysteine. Modification on Ser and Thr residues through a thioether bond was further confirmed by the identification of modification by GSH as well as Cys and homocysteine. Based on both accurate mass (within 5 ppm) and manual interpretation of tandem mass spectra, 36 different sites on 15 human lens proteins were identified as sites modified by thiol compounds (Table 1; (iii) in Fig. 1). Among them, 26 sites were modified by GSH, 17 sites were modified by Cys-Gly, and 13 sites were modified by cysteine. Only one site modified by homocysteine was identified perhaps reflecting the native concentrations of free homocysteine. In addition to thiol groups, lysine was also found to modify four proteins at four of the same sites as thiol modification, suggesting that a DHA intermediate is formed. Histidine modification of Ser or Thr residues was not observed. All of the modification sites reported in this paper except T170 in αB-crystallin and S34 in MP20 have been confirmed as phosphorylation sites in previous publications of the lens phosphoproteome (Huang *et al*., 2010; Wang *et al*., 2013). These results confirm a previous report that DHA can be formed from Ser or pSer residues under physiological conditions and can react with free thiol or amine groups (Masters, 1985). Importantly, the exact sites of modification have not been identified previously. In addition to Ser and pSer, DHA can also be formed from cysteine residues through the loss of H_2_S (Bar-Or *et al*., 2008). Endogenous DHA formation and GSH modification of a Cys residue were observed on Cys 5 in βA4-crystallin (Fig. S1). Similar modifications to those shown in Table 1 were observed in the water-soluble fraction (WSF) and urea-soluble fraction (USF).

**Table 1:** Modification sites identified in normal human lenses

	Sites that are modified by				
Proteins	GSH	Gly-Cys	Cys	Homo Cys	Lys
Beta-crystallin A3	S51, S59,S200	S59, S200	S59		S200
Beta-crystallin B2		S148	S148		S148
Beta-crystallin B1	T47, S97, T248	S97, S189, S190	S81		S81
Alpha-crystallin A	S13, S20, S59, S62, T148, S162	S59	S20, S59	S59	
Alpha-crystallin B	S53, S59, T170†, S138/S139	S59	S59		
Gamma-crystallin D		S40			S40
Gamma-crystallin D	S488, S607	S607			
Gamma-crystallin S	S90	S90, S172	S90		
Catenin beta-1	T551/S552				
Limbic system-associated membrane protein	S91	S91	S91		
Junctional adhesion molecule			S110		
Beta-crystallin A2		S146/S147	S146/S147		
MP20	S34†	S170	S170		
AQP0	S231, S235, S245	S229, S235	S229, S235		
Connexin 50	S259				

† Sites not previously reported as phosphorylation sites.

### *In Vitro* formation of DHA and glutathionylated peptides

To study whether the postulated two-step reaction, DHA formation followed by thiol addition, can occur spontaneously *in vitro*, and to unambiguously confirm the structures of the products, a modified αB-crystallin peptide containing phosphorylated Ser59 [(Ac-YAP(pSer)W); (i) in Fig. 1] was synthesized with the N-terminus of the peptide acetylated to prevent reaction with the alpha amino group. The peptide was incubated in 100 mm phosphate buffer (pH 7.4) to study the formation of DHA from phosphoserine. After 3 weeks at 60 °C, an Ac-YAP(DHA)W peak could be clearly detected by HPLC as shown in Fig. 3A. The Ac-YAP(DHA)W peptide was purified by HPLC (Fig. 3B), and its structure was confirmed by NMR (Fig. S2). This peptide represents a product of type (ii) in Fig. 1. Purified Ac-YAP(DHA)W was then mixed with either GSH or phenylethylamine in 100 mm phosphate buffer (pH 7.4) and incubated at 37 °C. Both phenylethylamine and GSH reacted readily with the DHA residue at 37 °C (Fig. 3C,D, respectively). Diastereoisomers were detected, as expected, for both the GSH and phenylethylamine products. The structures of both the GSH- and phenylethylamine-modified peptides were confirmed by MS/MS analysis, and the GSH-modified peptide is a type (iii) product in Fig. 1. Importantly, a significant HPLC peak due to the formation of Cys-Gly adducts was observed in the glutathione incubation (Fig. 3D). This result suggests that loss of glutamate from the GSH peptide adducts can occur spontaneously at neutral pH and is a natural degradation product of the GSH-modified peptide. The time course of formation of the GSH-modified peptide from purified Ac-YAP(DHA)W is shown in Fig. 3E. The level of the modified peptide increased over a period of hours at 37 °C reaching a plateau after 20 h. Correspondingly, the concentration of the DHA peptide diminished over the 24-h period and then remained steady (data not shown). The formation of a plateau is most likely due to the loss of GSH from the reaction mixture by conversion to its oxidized GSSG form. A second model phosphopeptide, aquaporin-0 (AQP0) 224-241 containing pSer 235, was incubated at 37 °C with 3 mm GSH, the concentration of GSH in cortical lens tissue. After incubation for 3 days, a GSH-modified AQP0 peptide at S235 was detected after Lys C digestion that was indistinguishable from the modified peptide detected in lens samples (Fig. S3).

### The level of glutathionylation of αB S59 in the different regions of the human lenses

**Figure 4:**
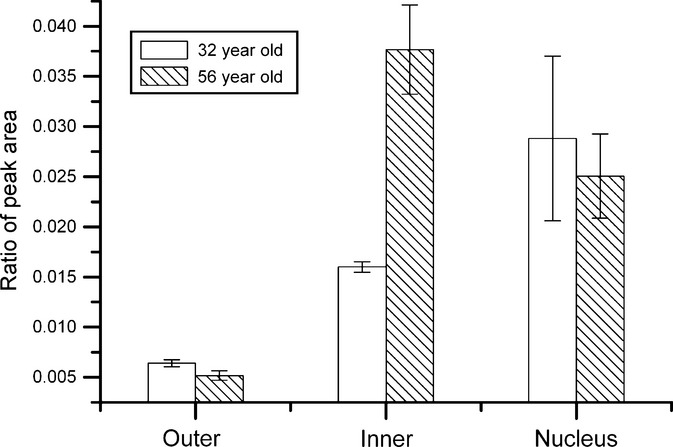
The level of glutathionylation of αB-crystallin S59 in the combined water-soluble and urea-soluble fractions from different regions of human lenses: The relative level of glutathionylation of S59 of αB-crystallin 57-69 relative to the level of unmodified peptide was measured in outer cortex, inner cortex, and nucleus regions of two human lenses (32 and 56 years old). Note that the ratio of the top six fragment ion intensities for each peptide are reported and, due to different fragmentation patterns, the ratios do not reflect the absolute ratio of peptide abundances. Error bars represent standard deviation calculated from replicate injections (*n* = 2) of the same samples.

The relative level of glutathionylation on αB S59 in the combined water-soluble and urea-soluble fractions in two human lenses of different ages was monitored, and the results are shown in Fig. 4. The results indicate that GSH modification (iii) is present in the young fiber cells of the outer cortex and the ratio of modified to unmodified peptide increases in the inner cortex in both lens samples. In the younger lens, the level of glutathionylation continues to increase in the nucleus, indicating the accumulation of more modified protein with protein age. In the older lens, the level of glutathionylation decreased in the nucleus compared to the inner cortex. The decreasing signal of GSH-modified peptide relative to unmodified peptide implies that further modification of the GSH-modified peptide occurs with age. In an aged (94-year-old) lens nucleus, the ratio of GSH-modified peptide to unmodified peptide is very low (Fig. S4), supporting the hypothesis that further degradation/modification of the GSH-modified peptide occurs with age. The effects of GSH concentration, protein age, and protein degradation in each lens region combine to produce the observed GSH-modified peptide abundance.

### Identification of crosslinked peptides from a cataractous lens

**Figure 5:**
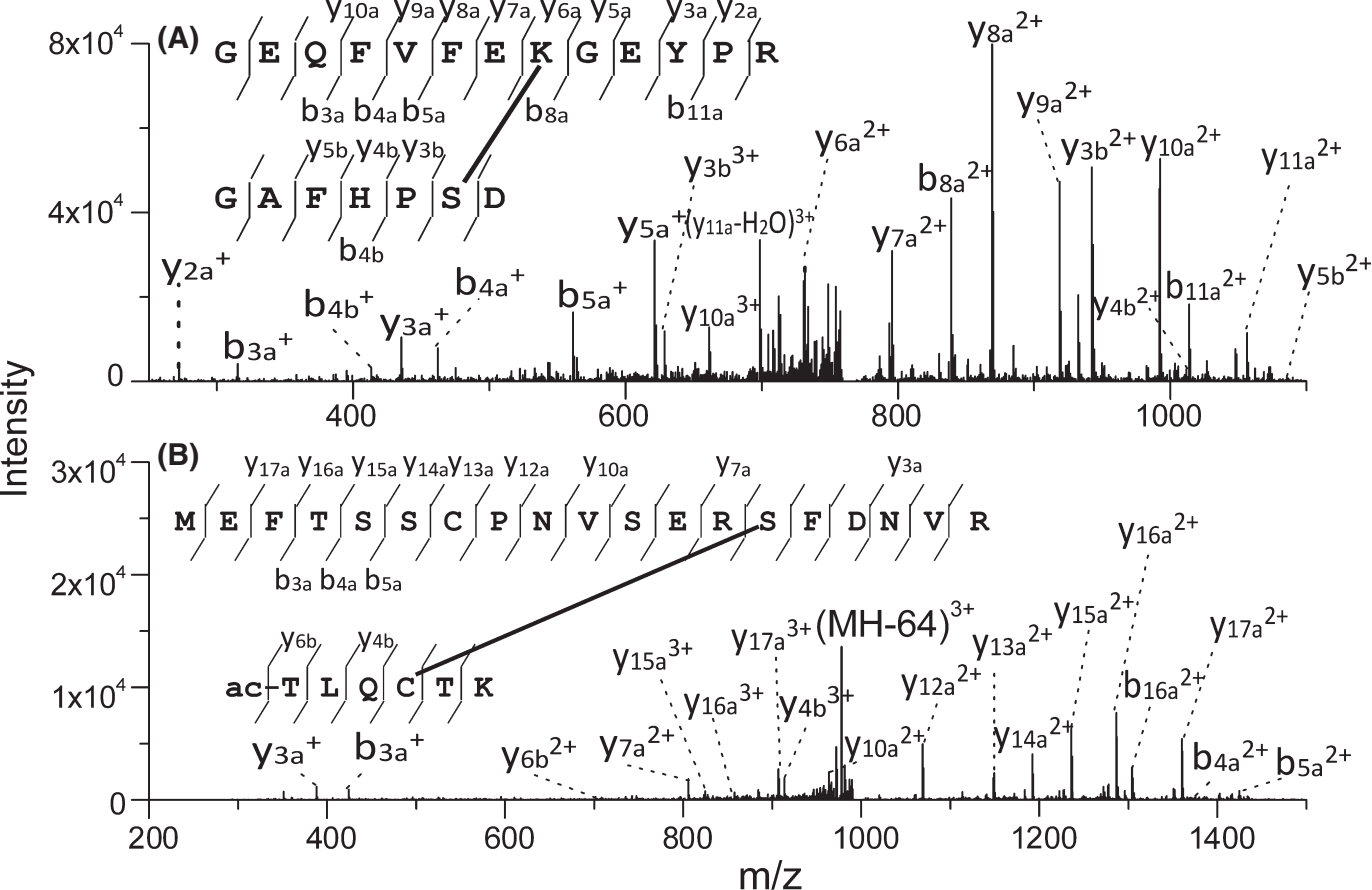
Tandem mass spectra of crosslinked peptides identified in a cataract lens nucleus: Tandem mass spectra of two crosslinked peptides are shown and observed fragments annotated for each peptide chain as a- or b-chains. (A): Crosslinked peptide between βB2 69-81: GEQFVFEKGEYPR (a) and βB2 198-205: GAFHPSN (b) through K76 and S204. (B): Crosslinked peptide between βA3 46-64: MEFTSSCPNVSERSFDNVR (a) and βA4 2-7: acetyl-TLQCTK (b) through S59 and C5.

Based on the identification of the thiol modifications described above, and reports of LAN, HAL, and LAL in lenses, we hypothesized that DHA and DHB intermediates could also lead to nondisulfide protein–protein crosslinks in aged lenses [products (iv) in Fig. 1], a phenomenon associated with human nuclear cataract formation (Truscott & Augusteyn, 1977a). Because the levels of HAL, LAN, and LAL were reported to be much higher in cataract lenses than in normal lenses (Bessems *et al*., 1987; Linetsky *et al*., 2004), crosslinked peptides were expected to be more easily detected in cataract lenses. Five crosslinked peptides corresponding to four sites of crosslinking were identified in the urea-insoluble fraction of the nuclear region of one human cataract lens as shown in Table 2, Fig. 5, and Fig. S5 (Supporting information). Assignment of crosslinked peptides was based on both accurate mass measurement and their tandem mass spectra.

**Table 2:** Crosslinked peptides identified in a cataract lens nucleus

Crosslinked peptides	[MH]^+^ calc.	[MH]^+^ obs.	Error (ppm)
βB2: GEQFVFEK†GEYPR	2297.0622	2297.0577	1.97
βB2: GAFHPS†N(deamination)			
βB1: AEFS†GECSNLADR	2152.9386	2152.9396	0.47
γS: EIHSC†K			
βA3: M(ox)EFTSSC(alkylated)PNVSERS†FDNVR	2994.3390	2994.3235	1.23
βA4: acetyl-TLQC†TK			
βA4: GFQYVLEC†DHHSGDYK	2598.1929	2598.1882	1.82
βA4: acetyl-TLQC†TK			
βA4: GFQYVLEC†DHHSGDYK	2325.0241	2325.0192	2.11
βA4: pQC†TK			

pQ, pyroglutamic acid.

All masses listed are monoisotopic masses.

† The residues that are involved in crosslinking.

One crosslinked peptide corresponds to a crosslink between two βB2-crystallin peptides 69-81 and 198-205. The tandem mass spectrum of this crosslinked product (Fig. 5A) indicates that residues K76 and S204 are involved in the crosslink. The identification of the peptides involved was deduced based on a series of b- and y-ions from peptide 69-81 and several b- and y-ions, as well as a strong y3 ion corresponding to N-terminal cleavage of the proline residue provided additional support for the presence of peptide 198-205. The tandem mass spectrum shown in Fig. 5B supports crosslinking between βA3-crystallin 46-64 and βA4-crystallin 2-7 peptides through residues Ser59 of βA3 and Cys5 of βA4 via a thioether linkage to Ser59.

Additional crosslinked peptides were observed as shown in Fig. S5 (Supporting information) where, in one case, a nondisulfide crosslink was observed between two cysteine residues with the loss of one molecule of H_2_S, suggesting DHA formation from one of the cysteine residues. The identification of this peptide crosslinked by a thioether bond, rather than a disulfide bond, was deduced based on sequence analysis of βA4 159-174 linked to βA4 2-7. Because peptide βA4 2-7 is short and N-acetylated, there were only two fragment ions above the noise level from this peptide; however, an additional crosslinked peptide (βA4 4-7 linked to βA4 159-174) was detected involving the same Cys 5 and Cys 166 residues of βA4-crystallin. The βA4 4-7 peptide could be generated from protein truncation that is widespread in the lens (Lampi *et al*., 1998; Grey & Schey, 2009; Su *et al*., 2012). Fig. S5B (Supporting information) indicates a crosslink between βB1-crystallin 74-86 and γS-crystallin 126-131 through residues Ser77 on βB1-crystallin and Cys130 on γS-crystallin. The strong neutral loss of 64 Da confirmed Met46 oxidation, and the y12 and y13 ions confirm carbamidomethylation of Cys52.

## Discussion

Beta-elimination of phosphate from phosphoserine and phosphothreonine occurs readily under basic conditions; however, as demonstrated in the current study, DHA can also arise from phosphoserine in peptides at physiological pH. DHA has been detected long-lived proteins such as dentin (Masters, 1985; Cloos & Jensen, 2000) and lens proteins (Srivastava *et al*., 2004), providing indirect evidence for serine or phosphoserine decomposition. Previously, Linetsky *et al*. reported glutathionylation of lens proteins presumably through nucleophilic addition to DHA (Linetsky & LeGrand, 2005); however, the ELISA technique used in that study could not identify the specific protein residues that were modified and could not confirm the type of bond formed. This current report confirms the nucleophilic addition of GSH, and its metabolites, to DHA residues in lens proteins and, for the first time, reports the identification of the proteins and residues that have been modified. Although DHA can be formed from either Ser or Cys, correlation of our data with lens phosphoproteomic data suggests that the majority of DHA in normal human lenses is produced by beta-elimination of phosphate from phosphoserine. Evidence for the formation of DHB from phosphothreonine and DHA from cysteine was also found. Previously, DHA has been reported to react with the imidazole group of His and the ε-amine group of Lys (Kanayama *et al*., 1987); however, we did not detect His modification and only detected a few sites modified by Lys, which could reflect the relatively low level of free His and Lys in the lens.

*In vitro* peptide incubations demonstrated that β-elimination of phosphoserine within a peptide to form a DHA residue, and the subsequent nucleophilic addition of either GSH or the Lys analogue, phenylethylamine, could occur spontaneously under physiological conditions (Fig. 3). Thus, proteins modified by thioether formation with GSH could be expected in long-lived proteins especially because crystallins present in the ocular lens are bathed in mm concentrations of GSH.

Glutathione plays a major role in the maintenance and regulation of the thiol redox status of most cells, but is particularly important in the lens (Lou, 2003) where there is a concentration gradient from almost 10 mm in the outer cortical cell layers to approximately 1 mm in the center of the lens (Harding, 1970). Increasing oxidative stress and decreased GSH levels are associated with nuclear cataractogenesis (Harding, 1970; Spector, 1995), and significant oxidation of protein is the feature that differentiates cataract lenses from aged-matched normal lenses (Truscott, 2005). It is important to distinguish the novel glutathionylation described in this report from the addition of GSH to proteins that occurs via disulfide bonds (Harding, 1970) and is a common reversible process that takes place when cells are exposed to oxidative stress. The irreversible glutathionylation process reported here would consume GSH in fiber cells and further decrease the concentration of GSH in the nucleus where the GSH synthesis and recycling pathways are presumed to be negligible (Rathbun, 1984). The functional consequences of the thiol modifications identified in this report are difficult to predict. Because the thiol modifications reported here occur on known phosphorylation sites, these age-related modifications could influence the structure and function of the modified proteins, particularly protein–protein interactions and perhaps cell signaling events that are regulated by phosphorylation. However, thioether modification by GSH also prevents DHA and DHB residues from further reaction to form protein–protein crosslinks that are likely to have more deleterious functional consequences. Thus, the irreversible thioether modifications may be protective against age-related protein aggregation.

The aggregation, crosslinking, and insolubilization of crystallins in the lens with age have been widely studied but remain poorly understood. A variety of post-translational modifications such as disulfide bonding (Truscott & Augusteyn, 1977b), glycation (Nagaraj *et al*., 1991), and transglutaminase-mediated crosslinking (Lorand *et al*., 1981) to crystallins could contribute to the protein aggregation, crosslinking and eventually lead to insolubilization. Crystallin aggregation and crosslinking are believed to form light-scattering centers and contribute to the development of cataract (Kanayama *et al*., 1987). A previous study characterized covalent multimers of crystallins from aging human lens by 2D-gel electrophoresis and mass spectrometry and reported two types of multimers including those containing eight different crystallins and others containing crystallins and beaded filaments (Srivastava *et al*., 2004); however, because as many as eight proteins were identified from a single gel spot, a question remains as to whether they all belong to a single covalently linked complex. In addition, detailed information on how and where these proteins are linked together remains unknown. In this report, we identified five crosslinked peptides in a 68-year-old cataractous lens nucleus that are presumably formed through DHA intermediates (ii).

For crosslinking to occur, the DHA (or DHB) intermediates (ii) and nucleophilic groups must be in close proximity to each other in the cell. Therefore, identification of crosslinked residues (iv) can provide important information on protein–protein interactions within the lens and potentially about the initiation and progression of protein aggregation (v). Amino- and carboxy-terminal regions in many proteins are commonly involved in the contacts between domains and subunits and stabilize the tertiary and quaternary structures by effectively tying the domains or subunits together (Thornton & Sibanda, 1983). The proximity of the crosslinked regions identified in this study could not be confirmed using currently available crystal structures because they do not provide distal N-terminal or C-terminal information. Four of the five protein–protein crosslinks identified in this study involve terminal regions. Moreover, all crosslinked peptides identified involve β-crystallins, a family of crystallins that are known to form dimers or oligomers. β-Crystallins contain an N-terminal extension and a C-terminal extension that are involved in the self-association to dimers and possibly higher oligomers (Mayr *et al*., 1994).

Using our current data, we could not distinguish whether crosslinks between identical protein subunits occur intermolecularly or intramolecularly. X-ray crystallography of βB2-crystallin has revealed a two-domain structure where the N- and C-terminal domains of one βB2-polypeptide are not in close contact, but are separated by an extended connecting peptide (Bax *et al*., 1990). Intriguingly the crystal structure showed a homodimer where the C-terminal domain of one βB2-crystallin subunit aligned next to the N-terminal domain of the other βB2-crystallin subunit (Bax *et al*., 1990). It seems very unlikely therefore that the crosslinked βB2-peptides involving residues 68-81 and residues 198-205 could come from a single βB2-subunit. This finding strongly suggests that βB2-crystallin exists as a homodimer in the intact lens. In a similar manner, βA4-crystallin is also reported to associate as a homodimer (Slingsby & Bateman, 1990), which may bring residues Cys5 and C166 in close proximity, intermolecularly, and, again, the crosslinking data provide evidence that this may be the form of the protein *in vivo*. The discovery of βB1-crystallin crosslinked to γS-crystallin (Fig. S4) confirms that these two proteins are packed closely together in the lens.

In summary, in normal lenses where GSH is abundant, the spontaneous generation of DHA and DHB residues (ii) in the long-lived structural proteins is most likely to result in irreversible GSH addition (iii) to the crystallins. Once GSH levels fall substantially, as they do in the earliest stage of nuclear cataract (Truscott & Augusteyn, 1977a), protein crosslinking (iv) is the inevitable result. Therefore, the thiol metabolite adducts (iii) may play a protective role in preventing protein–protein crosslinking (iv) and aggregation (v). This scenario is supported by results that show much higher levels of nondisulfide crosslinking of proteins from cataract than from normal lenses (Truscott & Augusteyn, 1977b; Linetsky *et al*., 2004). The fact that the extent of this novel covalent crosslinking increases significantly as the cataract worsens (Truscott & Augusteyn, 1977a) implies that this process and the mechanisms described in this paper could well play a key role in the formation of protein aggregates (v) leading to lens opacities.

In conclusion, this study, for the first time, identifies the sites in proteins that have been irreversibly modified by GSH and its metabolite as well as crosslinked through DHA- and DHB-mediated nucleophilic addition reactions. Moreover, we demonstrate that both DHA formation and nucleophilic addition can occur spontaneously under physiological conditions. Phosphoserine and phosphothreonine residues therefore are susceptible sites for spontaneous breakdown in long-lived proteins. Thus, the results presented here provide an important contribution to our understanding of protein aging, protein–protein aggregation, and potential mechanisms of cataract development. Furthermore, it is likely that similar modifications and protein–protein crosslinking reactions also take place in other long-lived cells, for example those in brain, cardiac muscle, and skin. The strategy and methods reported in this paper can be applied to search for similar modifications and crosslinking in other tissues.

## Experimental procedures

### Lens protein fractionation and trypsin digestion

Frozen human lenses (aged 32, 56, 58, 68 cataract, and 94 years) were obtained from NDRI (Philadelphia, PA, USA). A half human lens was decapsulated and homogenized in homogenizing buffer containing 25 mm Tris (pH 8), 5 mm EDTA, 1 mm DTT, 150 mm NaCl, 1% (v/v) phosphatase inhibitor (Sigma P5726, St. Louis, MO, USA), and 1% protease inhibitor (Sigma P8340). Lens proteins were then fractionated into WSF, USF, and urea-insoluble fraction (UIF) as previously described (Wang, *et al*., 2010) except that centrifugation at 100 000 *g* was used. Reduction using DTT and alkylation using iodoacetamide were accomplished as previously described (Wang *et al*., 2010). The UIF was centrifuged at 100 000 *g* for 20 min to remove excess iodoacetamide followed by a water wash. WIF and USF were filtered using a 10-kDa MWCO (Millipore, Billerica, MA, USA) filter to remove the protease inhibitor and urea. All samples were resuspended in 100 μL of 10% ACN in 50 mm ammonium bicarbonate buffer (pH 8.0). Sequencing-grade modified trypsin (enzyme/substrate = 1/500, Promega, Madison, WI, USA) was added and samples were incubated at 37 °C for 18 h. After digestion, samples were centrifuged at 20 000 *g* for 10 min, and the supernatant was collected and the remaining pellets were extracted by ACN (0.1% formic acid). The ACN extract was dried by SpeedVac and reconstituted in 5% ACN in 0.1% formic acid. The ACN extracts were pooled together with the supernatant and diluted fivefold by 0.1% formic acid and loaded onto a Sep-Pak C18 cartridge (Waters Corporation, Milford, MA, USA). The cartridge was washed with 0.1% formic acid and the bound peptides were eluted by 75% ACN (0.1% formic acid). Samples were then dried in a SpeedVac and stored at −20 °C until further analysis.

### Crosslinked peptide enrichment and identification

To identify crosslinked peptides formed *in vivo*, the lens nucleus was isolated from a quarter of a cataract lens (68 years old). The lens membrane fraction was prepared according to the methods described above. Tryptic peptides were extracted from the membrane pellets as described above, resuspended in 50 mm ammonium bicarbonate buffer (pH 8.0), and incubated with 1 μg of trypsin for another 18 h at 37 °C to reduce the number of missed cleavages. Tryptic peptides were reduced a second time in 10 mm TCEP at room temperature for 30 min followed by alkylation with 50 mm iodoacetamide at room temperature for 45 min. to insure complete reduction/alkylation. The peptides were cleaned up using a Sep-Pak C18 cartridge as described above. The sample was dried in a SpeedVac and tryptic peptides were offline-fractionated by strong cation exchange as described previously (Wang & Schey, 2011). Briefly, bound peptides were step-eluted sequentially from SCX resins by 40%, 60%, and 100% buffer B (5 mm potassium phosphate buffer containing 30% ACN, 350 mm KCl, pH 2.5) balanced with buffer A (5 mm potassium phosphate buffer containing 30% ACN, pH 2.5). The 60% buffer B eluate was dried in a Speedvac and reconstituted in 0.1% formic acid. The peptides were desalted using a C18 Ziptip (Millipore, Billerica, MA, USA) and eluted in 70% ACN (0.1% formic acid). The eluate was dried in a Speedvac and reconstituted in 0.1% formic acid and loaded onto a custom-packed SCX trap column (6 cm × 150 μm, Jupiter C18, 5 μm, 300 Å). Twenty microliters of 750 mm ammonium acetate was flowed through the column, and the remaining bound peptides were eluted by 30 μL of 3 m ammonium acetate, and the 3 m ammonium acetate eluate was used for crosslinked peptide identification by LC-MS/MS.

### *In vitro* formation of DHA and glutathionylated peptides:

To confirm that irreversible glutathionylation can occur spontaneously under physiological conditions, *in vitro* reactions were performed using lens protein-related peptides. A human AQP0 phosphopeptide 224-241 (phosphorylation on S235) was synthesized (MUSC Biotechnology Core Facility). A solution containing a final concentration of 0.5 mg mL^−1^ AQP0 peptide, 3 mm glutathione, and 5 mm TCEP in 100 mm phosphate buffer (pH 7.4) was prepared. The mixture was bubbled with nitrogen and incubated at 37 °C for 3 days. After 3 days, 10 μL of the sample was mixed with 1 μL of 0.5 mg mL^−1^ of endoproteinase LysC and the sample was incubated at 37 °C for 6 h and diluted tenfold by 0.1% formic acid. Lys C digestion was used to generate the same peptide detected in human lens digests and analyzed by 1D-LC-MS/MS.

In addition, an N-terminal acetylated peptide containing a phosphorylated Ser derived from the sequence of alpha B-crystallin [Ac-YAP(pSer)W] was synthesized (GLS Biochem, Shanghai, China). The peptide was incubated in 100 mm phosphate buffer pH 7.4 at 60 °C to promote the formation of DHA. Ac-YAP(DHA)W was purified using HPLC, and the formation of DHA was confirmed by mass spectrometry and NMR spectroscopy. Ac-YAP(DHA)W was incubated with GSH or phenylethylamine in 100 mm phosphate buffer pH 7.4 at 37 °C. The glutathionylated peptide product was again purified by HPLC and its structure confirmed by mass spectrometry. The time course of glutathionylation of the peptide was studied by incubating the purified Ac-YAP (DHA)W with GSH at 37 °C.

### The level of glutathionylation on S59 of αB-crystallin in different regions of the lens

To study the level of glutathionylation in the different regions of the lens, two human lenses (32 and 56 years) were dissected into outer cortex (about 0.5-mm-thick layer from the surface), inner cortex (about 1-mm-thick layer), and nucleus regions. The water-soluble and urea-soluble fractions were mixed together. The total protein per sample (500 μg) was reduced, alkylated, and digested as described above. Aliquots (0.25 μg) of each sample were analyzed by targeted LC-MS/MS *(pseudo* MRM) on LTQ Velos Pro. Both unmodified and GSH-modified αB 57-69 peptides were targeted by selecting their respective precursor ions and monitoring their fragment ion intensities in the MS/MS experiment. The integrated peak areas of top six transitions were calculated using Skyline software (MacLean *et al*., 2010). The total peak area of glutathionylated peptide was normalized by that of the unmodified peptide. Two injections were made per lens section.

### LC-MS/MS

Tryptic peptides were either separated on a one-dimensional fused silica capillary column (150 mm × 75 μm) packed with Phenomenex Jupiter resin (3 μm mean particle size, 300 Å pore size) or analyzed by multidimensional LC-MS/MS. One-dimensional liquid chromatography was employed using the following gradient at a flow rate of 0.4 μL min^−1^: 0–10 min: 2% ACN (0.1% formic acid), 10–50 min: 2–35% ACN (0.1% formic acid), 50–60 min: 35–90% ACN (0.1% formic acid) balanced with 0.1% formic acid. The eluate was directly infused into an LTQ Velos mass spectrometer (ThermoFisher, San Jose, CA, USA) equipped with a nanoelectrospray source. To identify the sites of DHA- and DHB-mediated modification, trypsin digests of lens fiber cell membrane fraction prepared from a 37-year-old and 58-year-old lens were analyzed by an 11-step multidimensional LC-MS/MS method as described previously (Wang *et al*., 2012). For crosslinked peptide identification, the sample was analyzed using 3-step salt pulse gradient (1 m, 1.5 m, and 2 m ammonium acetate) over a biphasic (SCX, C_18_) column. All LC-MS/MS analyses were run on a Velos Orbitrap mass spectrometer (ThermoFisher, San Jose, CA, USA). Dynamic exclusion (repeat count 2, exclusion list size 300, and exclusion duration 60 s) was enabled to allow the detection of less-abundant ions for all LC-MS/MS analyses.

### Data analysis

Tandem mass spectra were analyzed using a suite of custom-developed bioinformatics tools. All MS/MS spectra were converted to mzML files by Scansifter and searched on a 2500 node Linux cluster supercomputer using a custom version of the TagRecon algorithm (Dasari *et al*., 2010). Trypsin specificity was used with a maximum two missed cleavage sites. To identify unknown post-translational modifications, the data were searched against a custom human lens protein database using the TagRecon Blind searching algorithm. Once the mass shifts were identified, the data were then re-searched with differential modifications as well as a static modification of carbamidomethylation of cysteine and variable modification of oxidation of methionine and deamination of asparagine and glutamine. The identification of each modification reported required < 5 ppm mass accuracy and manual verification of the tandem mass spectra. In addition, a neutral loss of 129 due to facile loss of pyroglutamic acid was required to identify glutathionylated peptides.

For identifying crosslinked peptides, raw data were converted to MGF files by Scansifter, and crosslinked peptides were identified based on the combination of manual interpretation, analysis by Proteome Discoverer 1.1.0 (ThermoScientific, San Jose, CA, USA), a TagRecon blind search, and the MassMatrix algorithm (Xu *et al*., 2008). MS data were searched with mass tolerance of 5 ppm and a maximum mass error of 0.5 μ for MS/MS data. Crosslinks between Ser/Thr and Lys/His/Cys were searched using a mass shift of −18.01051 Da due to the elimination of H_2_O during crosslink formation. The possibility of formation of DHA from cysteine residues via the loss of H_2_S was also considered.

## Supporting Information

**Fig. S1** Selected ion chromatograms and tandem mass spectra of GSH-modified lens crystallin peptides. **Fig. S2** NMR spectra of synthetic peptides and reaction products. **Fig. S3** *In vitro* formation of glutathionylated AQP0 peptides. **Fig. S4** Extent of GSH modification on αB-crystallin S59 in combined water-soluble and urea-soluble fractions in distinct lens regions measured by MRM. **Fig. S5** Tandem mass spectra of crosslinked peptides identified in a cataract lens nucleus.
