# Supplementary material for: Human protein aging: modification and crosslinking through dehydroalanine and dehydrobutyrine intermediates
Source: Aging Cell. 2013 Nov 19;13(2):226–34. doi: 10.1111/acel.12164 (PMC4114717; doi:10.1111/acel.12164)
Supplement: Supplementary file 1 — Fig. S1 Selected ion chromatograms and tandem mass spectra of GSH-modified lens crystallin peptides. Fig. S2 NMR spectra of synthetic peptides and reaction products. Fig. S3 In vitro formation of glutathionylated AQP0 peptides. Fig. S4 Extent of GSH modification on αB-crystallin S59 in combined water-soluble and urea-soluble fractions in distinct lens regions measured by MRM. Fig. S5 Tandem mass spectra of crosslinked peptides identified in a cataract lens nucleus. [file acel0013-0226-sd1.docx]

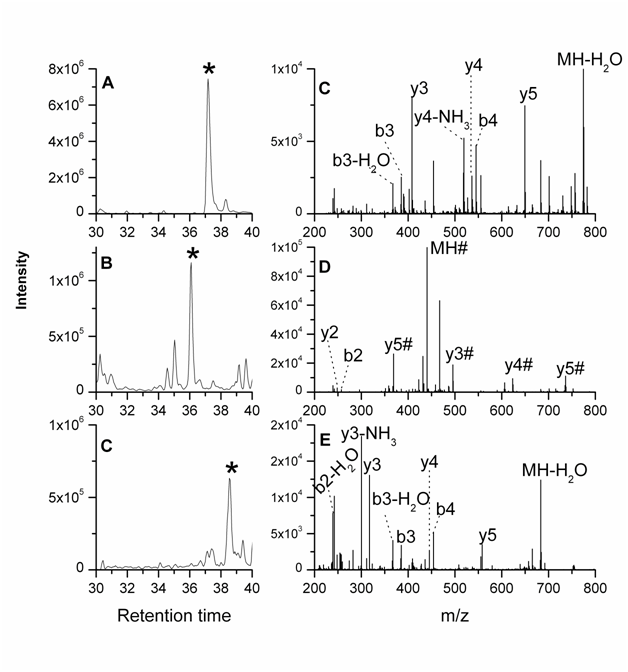


**Supplemental Figure 1**. Selected ion chromatograms for (A) unmodified βA4 2-7, (B) βA4 2-7 with Cys 5 modified by GSH, (C) βA4 2-7 with Cys 5 converted to DHA are plotted. Asterisks indicate the peak of interest. The tandem mass spectrum for each peptide is shown on the right side of its chromatogram. # indicates fragment ions with neutral loss of 129.


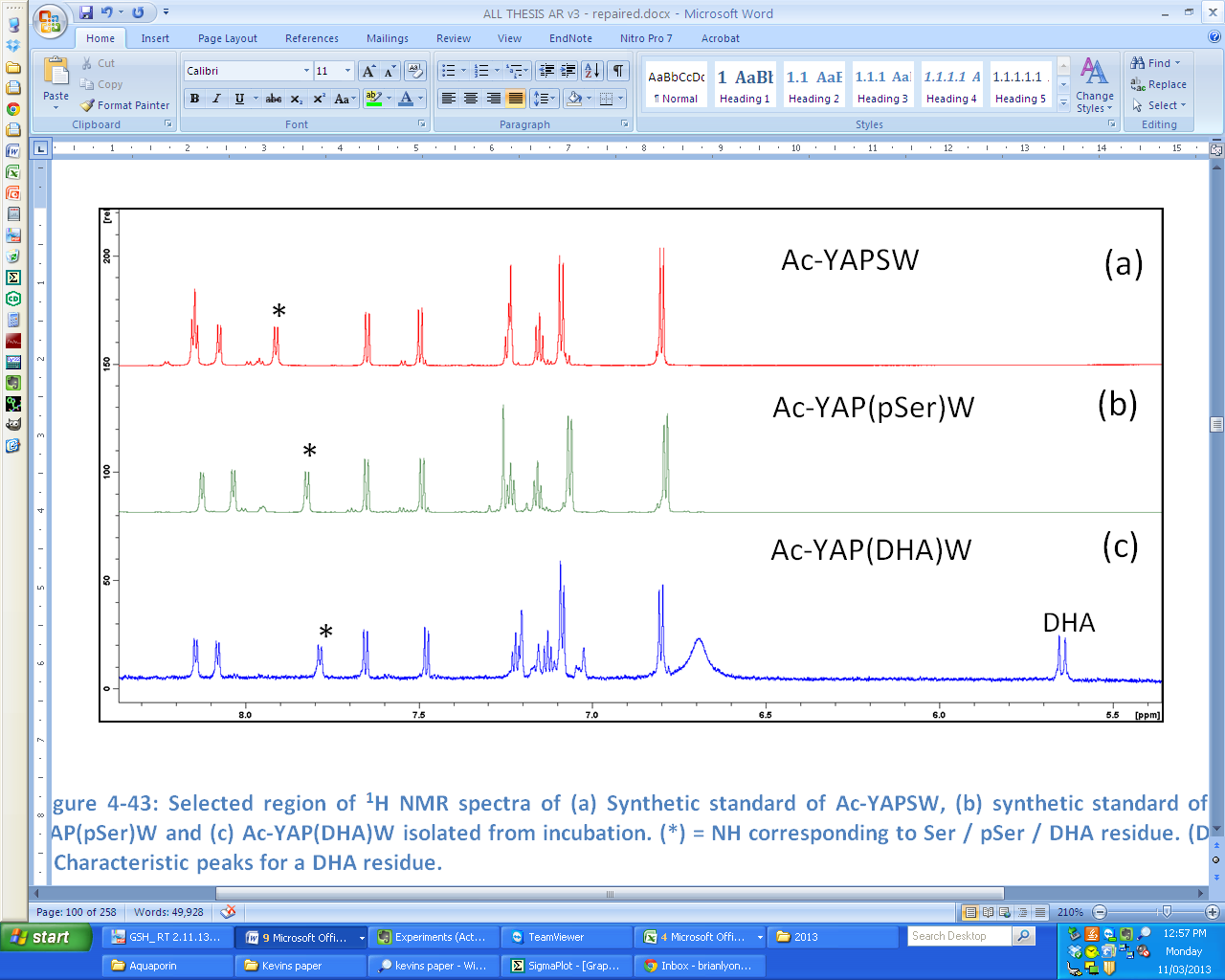


Supplemental Figure 2. Selected region of ^1^H NMR spectra of (a) Synthetic standard of Ac-YAPSW, (b) synthetic standard of Ac-YAP(pSer)W and (c) Ac-YAP(DHA)W isolated from incubation. (*) = NH corresponding to Ser / pSer / DHA residue. (DHA) = Characteristic peaks for a DHA residue.

#



**Supplemental Figure 3:** *In vitro* formation of glutathionylated AQP0 peptides. Phosphorylated peptide AQP0 224-241(phosphorylation on S235) was incubated with 3 mM GSH at 37 ^o^C in 100 mM phosphate buffer (*p*H 7.4) for three days. After incubation, the peptide was digested by endoproteinase Lys C to yield AQP0 peptide 229-238. (A) Selected ion chromatogram of GSH modified AQP0 229-238 peptide indicating the formation of diastereoisomers (asterisks). (B) Tandem mass spectrum of glutathionylated AQP0 peptide formed *in vitro* and (C) tandem mass spectrum of modified AQP0 peptide detected in human lens sample. b- and y- ions are labeled and asterisks indicate fragment ions with neutral loss of 129.

**

**

Supplemental Figure 4. Targeted quantitation of aB-crystallin peptide 57-69 in three lens regions. The ratio of the top six fragment ion intensities for GSH-modified and unmodified peptides are shown for three lens regions from three different lens ages.

#

Supplemental Figure 5: Tandem mass spectra of crosslinked peptides identified in a cataract lens nucleus: (A): Crosslinked peptide between βA4 159-174: GFQYVLECDHHSGDYK (a) and βA4 2-7: acetyl-TLQCTK (b) crosslinked through residues C166 and C 5. (B): Crosslinked peptide between βB1 74-86: AEFSGECSNLADR (a) and γS 126-131: EIHSCK (b) crosslinked through residues βB1 S77 and γS C130.
